# Malignant Transformation of Renal Cyst with Bosniak I Features

**DOI:** 10.3390/diagnostics15111326

**Published:** 2025-05-26

**Authors:** Sandra Ćulap, Filip Brkić, Andro Matković, Jelena Svetec, Nikolina Jurjević, Katarina Horvat Pavlov, Vinko Vidjak, Thomas Ferenc

**Affiliations:** 1 School of Medicine, University of Zagreb, 10000 Zagreb, Croatia; culapsandra@gmail.com (S.Ć.);; 2 Department of Diagnostic and Interventional Radiology, Merkur University Hospital, 10000 Zagreb, Croatianikolinajurjevic@yahoo.com (N.J.); 3 Department of Pathology, Merkur University Hospital, 10000 Zagreb, Croatia

**Keywords:** Bosniak classification, Bosniak I cyst, Bosniak IV cyst, cystic renal lesion, malignancy, renal cell carcinoma

## Abstract

The Bosniak classification categorizes renal cystic lesions based on cross-sectional imaging features from clearly benign (Bosniak type I) to malignant lesions (Bosniak type IV). A 67-year-old female patient presented to the emergency department with typical symptoms of acute cholecystitis. During a transabdominal ultrasound examination, an incidental finding was a suspicious cluster of anechoic cystic lesions with internal septa in the left kidney. Following contrast-enhanced computed tomography (CT), the lesion was categorized as a Bosniak type IV cyst. Compared to an earlier CT scan, a Bosniak type I cyst preceded the current Bosniak type IV cyst, suggesting a malignant alteration over the 7-year interval. It was surgically removed, and pathohistological analysis revealed cystic renal cell carcinoma. Although simple renal cysts rarely become malignant, scientific discussion about potential algorithms for additional surveillance is needed.

**Figure 1:**
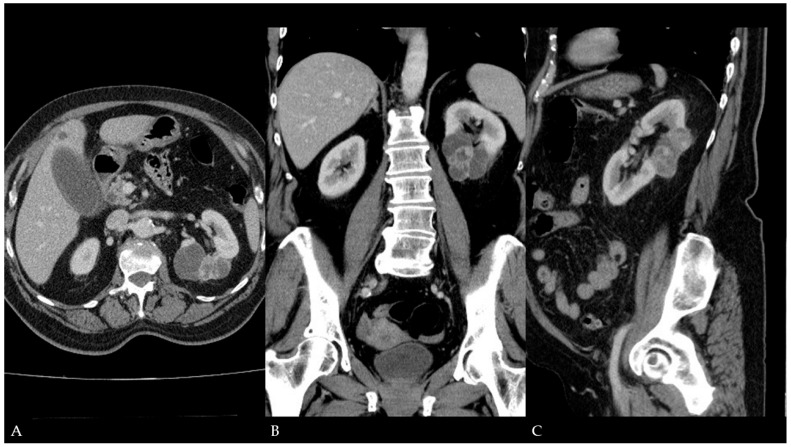
Contrast-enhanced computed tomography (CT) of the abdomen and pelvis (year 2022): (**A**)—Axial plane; a partially extracortical, macro-lobulated cystic lesion is seen in the lower pole of the left kidney, measuring 6.5 × 6.3 × 4.4 cm (measurement lines are not depicted) with thick internal septa and solid nodules enhanced following contrast media administration. (**B**,**C**)—Coronal and sagittal planes; the morphology of the depicted cystic lesion is indicative of a malignant renal lesion (Bosniak type IV cyst). A 67-year-old female patient presented to the emergency department with pain in the right upper abdominal quadrant after a large meal, followed by nausea and vomiting. The initial laboratory results revealed leukocytosis, elevated C-reactive protein (CRP), and liver enzyme levels (AST, ALT, and GGT). Her symptoms were typical clinical features of acute cholecystitis. The patient was referred for an abdominal ultrasound (US) evaluation, which demonstrated signs of acute cholecystitis with dilation of the common bile duct. However, the US examination also revealed an atypical conglomerate of anechoic cystic lesions with internal septa in the lower pole of the left kidney. A subsequent contrast-enhanced abdominal and pelvic CT scan demonstrated a macro-lobulated cystic lesion in the lower pole of the left kidney with thick internal septa and solid nodules. The septa and solid component showed contrast enhancement indicative of a Bosniak type IV cystic lesion (Figure 1). An earlier CT examination (from 2015) was available for comparison, with a Bosniak type I lesion at the exact location in the left kidney, measuring up to 8 cm in diameter (Figure 2). It was assumed that the Bosniak type I lesion had malignantly altered into a Bosniak type IV lesion. Due to the urgency, it was decided first to perform a cholecystectomy, while a radical nephrectomy was scheduled four months later. Pathohistological analysis confirmed the presence of predominantly cystic clear cell renal cell carcinoma (RCC) (WHO grade 3, pTNM, pT2a, pNx, pMx) (Figure 3). The patient was discharged home in good general condition. The follow-up US and CT scans in the 3-year interval revealed no disease recurrence. The Bosniak classification is a system used for categorizing renal cystic lesions based on cross-sectional imaging features, typically using contrast-enhanced CT or magnetic resonance imaging (MRI) [1]. Recently, contrast-enhanced ultrasound (CEUS)-adopted Bosniak classification was also introduced [2]. Bosniak type I and II cysts are considered benign and require no further follow-up, Bosniak IIF cysts are probably benign and follow-up is recommended, and Bosniak III cysts are probably malignant and, due to a high risk of malignant alteration, surgery or active imaging surveillance is advised, whereas Bosniak type IV lesions are considered malignant and require only surgical intervention [3]. RCC on imaging studies presents as a predominantly cystic mass in up to 22% of cases [4]. Bosniak type I cysts are typically benign and do not require additional monitoring. However, the malignancy rate in Bosniak I type cysts is reported between 1.7% and 3.2% [3,5]. In this case, an alteration into cystic RCC was described, prompting the question of whether additional surveillance is necessary for simple cysts even though they rarely develop into malignancies. Furthermore, many new diagnostic techniques have been designed to improve diagnostic accuracy in differentiating benign from malignant solid or cystic renal lesions, especially after introducing artificial intelligence (AI) [6,7], which may facilitate regular surveillance.

**Figure 2:**
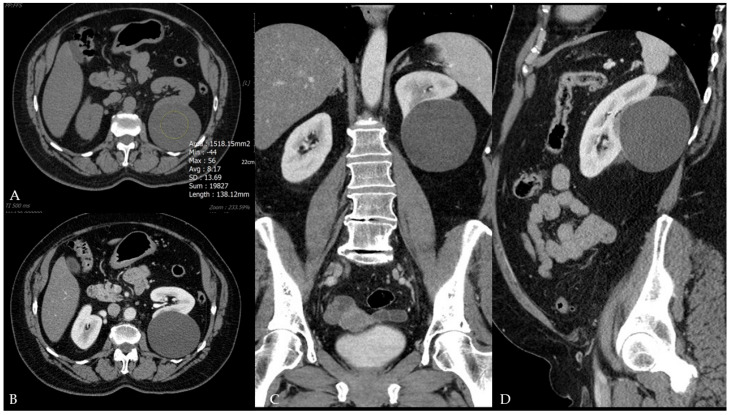
Computed tomography of the abdomen and pelvis (year 2015): (**A**)—Non-contrast-enhanced image in the axial plane; a large, round, and partially extracortical cystic lesion is seen in the lower pole of the left kidney, measuring up to 8 cm in diameter (measurement lines are not depicted) with no internal septa or solid component. The lesion contains water content [i.e., at the internally placed region of interest (ROI)] measuring around 8 Hounsfield units. (**B**)—A contrast-enhanced image in the axial plane; the same lesion demonstrates no enhancement following contrast media administration. (**C**,**D**)—Contrast-enhanced images of the same lesion in the coronal and sagittal planes confirm its completely benign morphology, correlating with a Bosniak type I cystic lesion.

**Figure 3:**
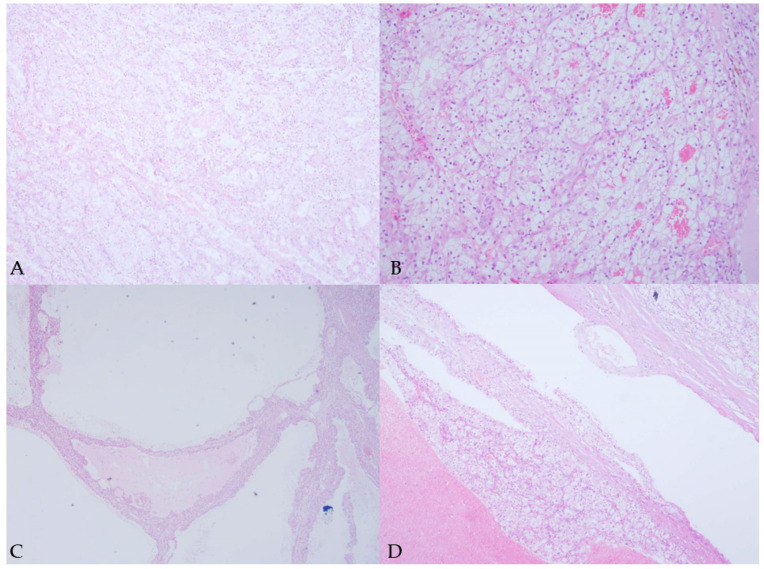
Pathohistological images of a surgically removed Bosniak type IV cyst. Pathohistological analysis confirmed the presence of predominantly cystic clear cell RCC (WHO grade 3, pTNM, pT2a, pNx, pMx), which, on a macroscopic level, did not penetrate the kidney capsule and did not infiltrate the surrounding fatty tissue. The renal artery, vein, and ureter appeared normal. (**A**)—H&E, magnification 20×, showing compact nests and alveolar formations of atypical epithelial cells with clear cytoplasm and distinct membranes. (**B**)—H&E, magnification 200×, demonstrating compact nests and alveolar formations of atypical epithelial cells with clear cytoplasm and distinct membranes. Nucleoli are conspicuous and eosinophilic at 100× magnification. (**C**)—H&E, 20×, showing pseudocystic parts of the tumor. (**D**)—H&E, 40×, also showing pseudocystic parts of the tumor.

## Data Availability

Not applicable.
